# Ciprofloxacin-loaded dissolving polymeric microneedles as a potential therapeutic for the treatment of *S. aureus* skin infections

**DOI:** 10.3762/bjnano.13.43

**Published:** 2022-06-15

**Authors:** Sharif Abdelghany, Walhan Alshaer, Yazan Al Thaher, Maram Al Fawares, Amal G Al-Bakri, Saja Zuriekat, Randa SH Mansour

**Affiliations:** 1 School of Pharmacy, University of Jordan, Amman, 11942, Jordan; 2 Cell Therapy Center, University of Jordan, Amman, 11942, Jordan; 3 Faculty of Pharmacy, Philadelphia University, Amman, 19392, Jordan

**Keywords:** dissolving microneedles, microneedles, polyvinyl alcohol (PVA), polyvinylpyrrolidone (PVP), skin infection

## Abstract

Microneedles have been widely studied for many topical and transdermal therapeutics due to their ability to painlessly puncture the skin, thereby bypassing the stratum corneum, the main skin barrier. In this study, ciprofloxacin (CIP) was loaded into dissolving polymeric microneedles prepared by a two-layer centrifugation method as a potential treatment of skin infections such as cellulitis. The polymers used were polyvinyl alcohol (PVA) and polyvinylpyrrolidone (PVP). Two formulations were investigated, namely CIP_MN1, composed of 10 mg ciprofloxacin incorporated into a polymer matrix of PVA and PVP with a weight ratio of (9:1), and CIP_MN2, composed of 10 mg ciprofloxacin incorporated into PVA polymer. CIP_MN1 and CIP_MN2 showed a mean microneedle height of 188 and 179 µm, respectively. Since Parafilm has been proven as a model to examine the perforation of microneedles in skin, it was used to evaluate the ability of microneedles to perforate the skin. CIP_MN1 showed almost complete perforation of Parafilm, 190 pores, compared to CIP_MN2 which created only 85 pores in Parafilm, and therefore CIP_MN1 was used for subsequent studies. Examining CIP_MN1 on agarose gel as an in vitro model of human skin showed that the formula was able to fully perforate the agarose gel. Moreover, this formula showed significantly greater antimicrobial activity (*p* < 0.0001) compared to a free gel of ciprofloxacin against *Staphylococcus aureus* in an agarose gel-based model. This was evidenced by a zone of inhibition of 29 mm for the microneedle formulation of ciprofloxacin (CIP_MN1) compared to 2 mm for the free gel of ciprofloxacin. Furthermore, the CIP_MN1 showed complete dissolution in human skin after 60 min from application. Finally, the skin deposition of CIP_MN1 was investigated in ex vivo excised human skin. CIP_MN1 showed significantly more deposition of ciprofloxacin in deeper skin layers compared to the free gel of ciprofloxacin, and the released ciprofloxacin from the microneedles tends to migrate to deeper layers with time. Collectively, these results suggest that CIP_MN1 can be a potential delivery system for the treatment of *S. aureus* skin infections.

## Introduction

Topical and transdermal drug delivery is a major route for the administration of antimicrobials to the infected parts of the skin and to the systemic circulation. The main limitation to dermal drug delivery is skin barriers. This is mainly due the uppermost dead keratinized skin layer known as the stratum corneum [1]. Microneedles of different shapes and sizes have been utilized to overcome this limitation since they can painlessly penetrate the upper skin layers [2]. Patients can self-administer microneedles and, thus, overcome the pain associated with conventional parenteral injections. Moreover, this drug delivery system can potentially overcome the low bioavailability of orally administered drugs due to poor absorption and enzymatic degradation in the gastrointestinal tract [3–4].

Among the different types of microneedles, dissolving microneedles have attracted research due to their advantages, which include low cost and simple preparation techniques. These microneedles are manufactured of polymers incorporated with medicaments and are intended to dissolve completely in the skin, permitting the medicament to be distributed in deeper skin layers to treat local and systemic infections [5].

Previous studies have shown the advantages of dissolving microneedles in reducing the microbial burden in bacterial and fungal skin infections [6–7]. Herein, we are aiming at providing a potential microneedle delivery system for the treatment of staphylococcal skin and soft tissue infections such as cellulitis. Cellulitis, as an example of common bacterial skin infection, is a potentially serious disease that involves the dermis and subcutaneous tissues [8]. Gram-positive cocci such as *Staphylococcus aureus* are among the predominant causes of cellulitis [9–11]. Ciprofloxacin is a broad spectrum quinolone antibiotic [12], reported to effectively manage soft tissue and skin infections (STSI) caused by *S. aureus* [13–16]. However, the therapeutic dose of ciprofloxacin upon using conventional delivery systems is relatively high and associated with adverse effects, which partially contributed to its current limited use in the management of *S. aureus* STSI [17].

Therefore, a proper delivery system that can evade the barrier properties of the skin is essential to be effective in the treatment of cellulitis and other skin infections. In this work, the potential of dissolving polymeric microneedles loaded with ciprofloxacin for the treatment of *S. aureus* skin infections was investigated. Recent studies showed that dissolving polymeric microneedles are a promising approach in topical and transdermal drug delivery because of the rapid dissolution and/or degradation of the polymer, which, in turn, releases the incorporated drug [18]. These dissolving microneedles are based on polymers such as PVA, PVP, chitosan, and poly(lactide-*co*-glycolide) [19].

In our study, polymer-based microneedles were optimized to achieve a formulation that can efficiently penetrate the skin. In this context, microneedles were studied regarding drug content and penetration of Parafilm and an agarose-based skin model. Parafilm has been utilized to test the insertion properties of polymeric microneedles [20]. Parafilm-M is mainly composed of paraffin waxes and polyalkene (polyolefin) [21] and, thus, presumably similar in hydrophobicity to the stratum corneum, the main barrier of human skin. Also, the dissolution of these microneedles in excised human skin and the concentration of ciprofloxacin in each layer of the excised human skin was studied. We designed an in vitro model of skin infection to compare the microneedles with free gel and, consequently, the antimicrobial activity of ciprofloxacin-loaded polymeric microneedles against *S. aureus*. Agarose gel, a transparent gelatinous substance composed of a carbohydrate polymer extracted from certain red seaweed, was proposed in recent studies as an in vitro model for the mechanical properties of the human skin [22–23]. Gelatinous substances can interact with water, which allows one to control various physical, mechanical, and chemical properties of the artificial skin, such as hardness, and surface properties [24]. Therefore, agarose gel was utilized as artificial skin and was subsequently treated with inoculum of *Staphylococcus aureus* to study the antibacterial activity of ciprofloxacin-loaded polymeric microneedles.

To assess the distribution of CIP in dermal layers after microneedle application, the cryostatic microtome technique was utilized. This technique is based on freezing excised skin samples and slicing them using a microtome for microscopic examination and analysis. The optimal critical temperature (O.C.T) compound is used to stabilize the skin during sectioning below −10 °C [25–27]. The sliced layers were analyzed for CIP content in a similar manner to that described in [25].

## Experimental

### Materials

Ciprofloxacin hydrochloride, PVA, PVP, acetonitrile, phosphoric acid, triethylamine, and phosphate buffer tablets were purchased from Merck, Germany. *Staphylococcus aureus* (ATCC-29213) was purchased from ATCC, USA. Poly(dimethylsiloxane) (PDMS) molds, including 196 laser-engineered pyramidal holes measuring 200 µm in depth and 50 µm base diameter were procured from Micropoint company, Singapore. Parafilm-M (thickness 0.13 mm) was purchased from Merck, Germany. HPLC-grade double-distilled water was used. O.C.T compound clear was obtained from Agar Scientific, UK.

### Analysis of ciprofloxacin content in needles and skin

The analysis of ciprofloxacin was adopted from the United States Pharmacopoeia (USP) and was performed using HPLC (Shimadzu HPLC, model LC-2030C PLUS 3D). The system included an integrated solvent and degasser, an analytical pump, a thermostatic autosampler, a UV detector, and a thermostatic column compartment. Data acquisition was performed via the LabSolutions LCGC software. The eluent was detected at 278 nm. Separation was carried out using a reversed-phase Interclone C18 column (250 mm × 4.6 mm, 5 μm particle ODS 100 Å size) (Phenomenex, California, USA) at 30 °C. The mobile phase had an isocratic composition of 0.025 M phosphoric acid (pH 3.0 ± 0.1) previously adjusted with triethylamine/acetonitrile (80:20 v/v), eluted at a flow rate of 1.5 mL/min. The injection volume was 20 µL. The drug concentration was then determined according to a calibration curve. The calibration curve was plotted by correlating the peak area measured with known concentrations of ciprofloxacin samples. All measurements were conducted in triplicate to calculate mean values and standard deviations.

### Preparation of ciprofloxacin-loaded polymeric microneedles

**Figure 1:**
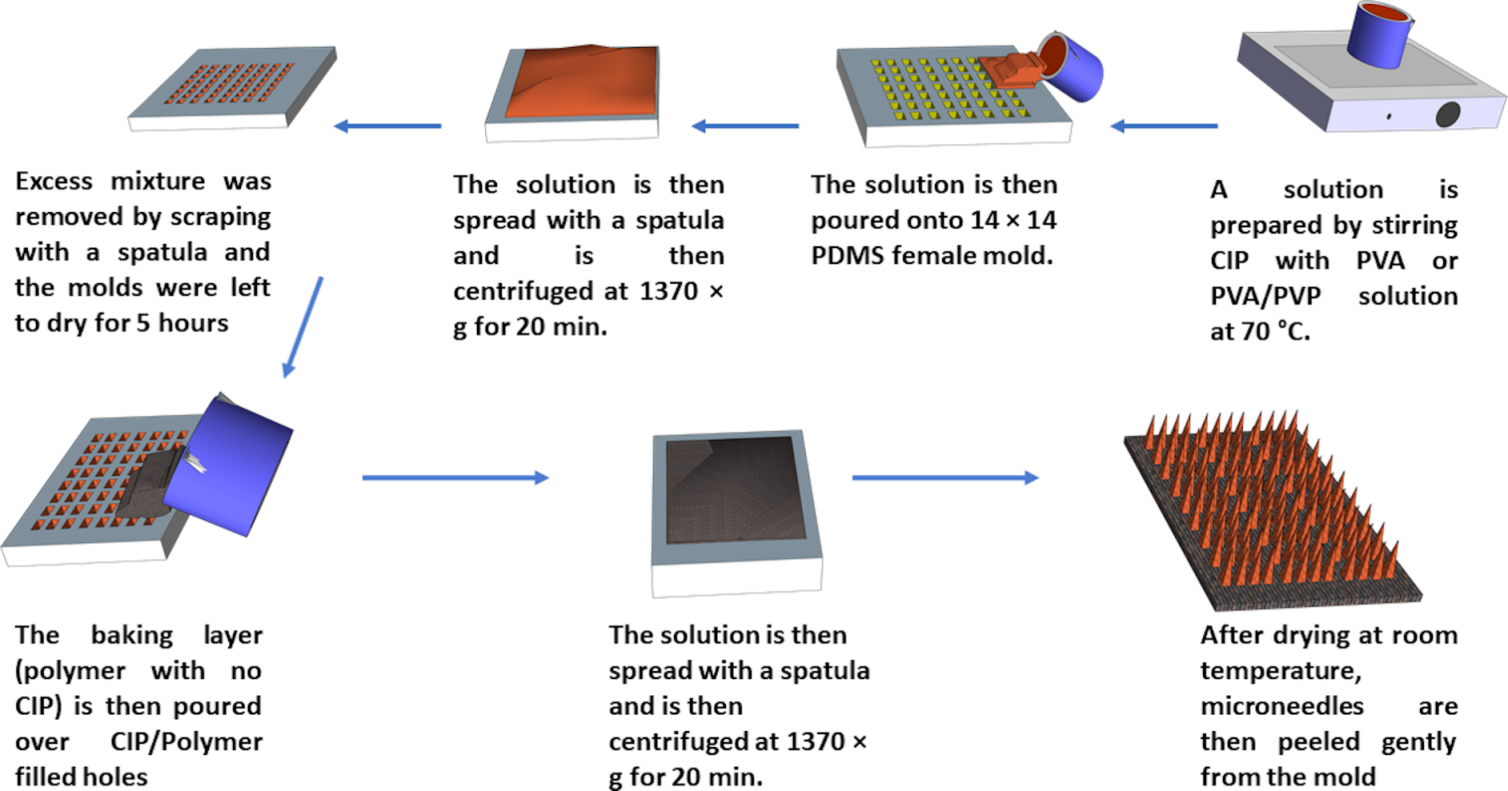
Microneedle fabrication using the two-layer centrifugation method. PVA/PVP or PVA gel with ciprofloxacin is stirred at 70 °C and poured on 14 × 14 PDMS mold and spread with a spatula. The mold is then centrifuged to fill the mold holes. The baking layer, which is free of the drug, is then spread over the mold, centrifuged, and allowed to dry forming polymeric microneedles.

20% PVA (w/w) and 60% (w/w) PVP aqueous solutions were prepared by heating at 70 °C while stirring the solution with a magnetic stirrer (IKA-Werke GmbH & Co. KG, Staufen, Germany). Microneedles were prepared via a two-layer centrifugation method, as shown in Figure 1. Briefly, 10 mg CIP and 9 g of aqueous 20% w/w PVA/60% w/w PVP (9:1 weight ratio) or 9 g of 20% w/w PVA alone were stirred using a magnetic stirrer at 70 °C. Next, the mixture of CIP and PVA or PVA/PVP was centrifuged at 145*g* for 5 min (Eppendorf Centrifuge 5804, Merck KGaA, Darmstadt, Germany) to remove the air bubbles. The resulting mixture was then poured into 14 × 14, poly(dimethylsiloxane) (PDMS) molds (Micropoint company, Singapore). Each hole in the mold was 200 µm in depth and 50 µm diameter. The molds were then centrifuged at 1370*g* for 20 min to allow the polymer solution to fill the mold holes. Excess mixture was removed by scraping with a spatula and the molds was left for 5 h to dry. The aqueous baseplate solution of 20% w/w PVA (with no drug) was poured on the molds and centrifuged for 20 min at 699*g*. Next day, the dried microneedles were peeled from the mold gently. Blank microneedles were prepared in a similar manner without the addition of CIP to the PVA or PVA/PVP solution in the initial step.

### Microneedle characterization

The shape and dimensions of CIP_MN1 and CIP_MN2 arrays were examined using a digital light microscope (Swift SW380B, China). Studying the mechanical strength of the microneedles, heights before compression were first measured. After pressing the microneedle arrays by thumb for 30 s, the microneedle heights were measured again.

### Preparation of artificial agarose skin

Previously, agarose gel was utilized as an artificial skin model [28]. Briefly, 2.5 g of agarose was suspended in 100 mL water. The dispersion was then placed in the microwave to melt for 2 min. The solution is then loaded into a 6-well plate with a thickness of 2 mm and incubated at room temperature for 1 h to solidify. The gel was then removed from the wells and used to study the insertion properties and for in vitro infection assay.

### Preparation of excised human skin samples

The excised human skin specimen was obtained from Shmeisani hospital under ethical approval No. UPR. 101/6-45. The consent of the skin donor, a 38 year-old female, was verbally granted when undergoing abdominal plastic surgery. She had the option to dump the specimen or donate it for scientific research. Her consent was granted before and after surgery and was witnessed by the surgeon. The skin was obtained from Al-Shmeisani hospital immediately after the surgery and treated as previously obtained specimens [29]. Specimens were defatted using a scalpel, cleaned by tapping with dry wipes and placed on paperboard wrapped with aluminum foil with the skin surface facing upward. Then, the skin was covered with aluminum foil, kept in zipper plastic bags, and subsequently stored at −70 °C for a maximum period of 6 months.

### Microneedle dissolution in the skin

Prior to use, skin specimens were equilibrated in 1× phosphate-buffered saline (PBS), pH 7.4, for 15 min. A section of full-thickness human skin was placed, with the stratum corneum facing upward, onto a piece of tissue paper soaked with PBS in a weighing boat. CIP_MN1 were adhered to a piece of plastic tape and manually applied to the skin. To prevent the skin from drying out, another inverted weighing boat was placed on the top of the weighing boat containing the skin specimen and sealed with plastic tape. At predefined time points, CIP_MN1 were withdrawn from the skin and their heights were measured using the digital microscope.

### Skin deposition studies

**Figure 2:**
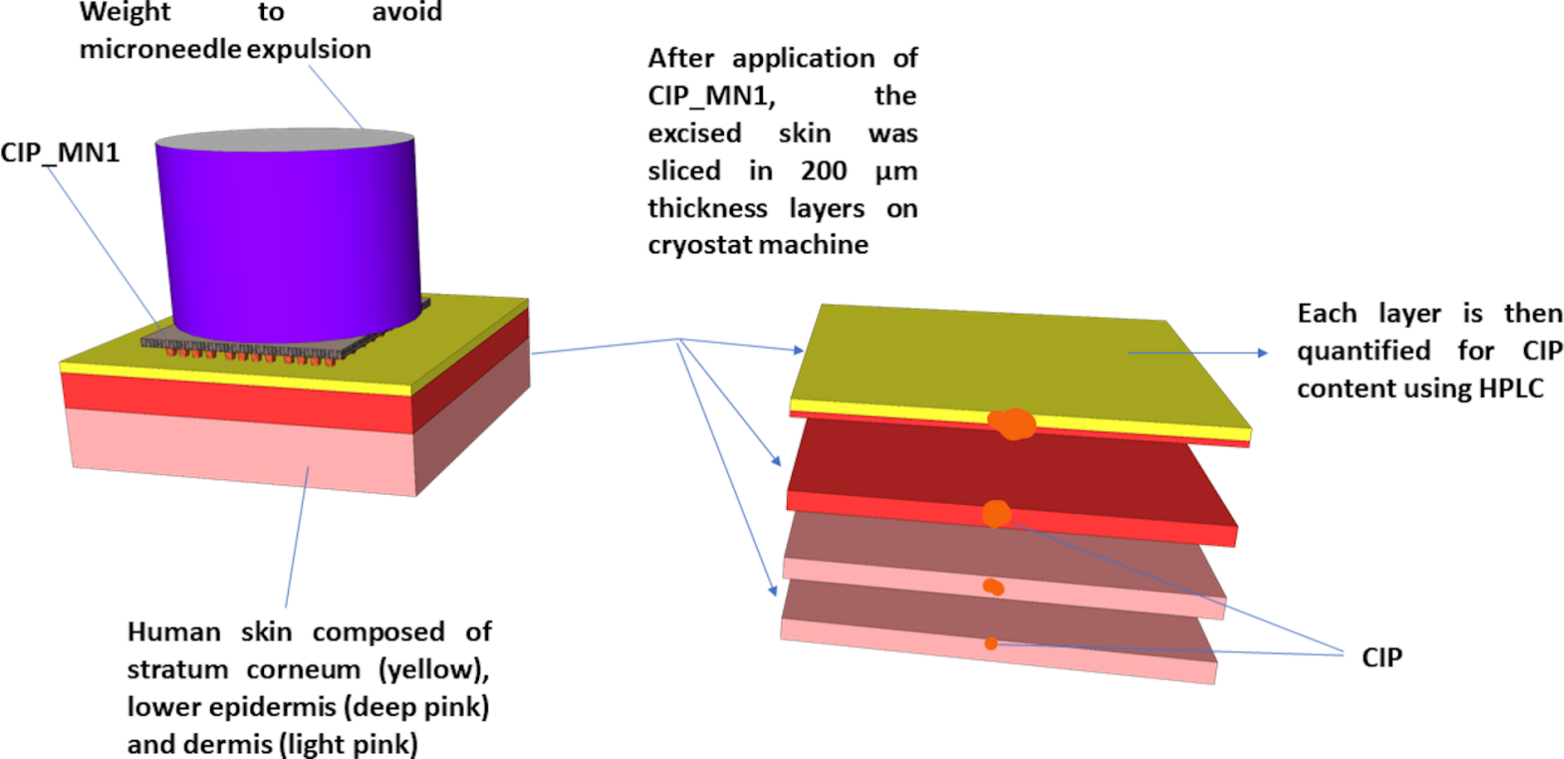
Schematic presentation of the skin deposition study of CIP_MN1. The full-thickness human skin was excised and treated. CIP_MN1 was then inserted into the skin using finger pressure applied to the microneedle baseplate for 30 s. A cylindrical 10 g stainless steel weight was placed on top of CIP_MN1 array to prevent expulsion of the microneedles, and the tissue paper was frequently wetted with PBS to prevent the skin from drying out. At each predefined time point, CIP_MN1 was removed, and the skin was sliced using a cryostatic microtome.

This study was conducted in a manner analogous to the procedure in [25]. Briefly, CIP_MN1 were inserted into the full-thickness human skin using finger press applied to the microneedle baseplate for 30 s. As shown in Figure 2, a cylindrical 10 g stainless steel weight was placed on top of the CIP_MN1 and the tissue paper was frequently soaked with PBS to prevent the skin from drying out. At each predefined time point, CIP_MN1 were removed, the skin was placed on dental wax and 1 cm^2^ of skin was excised using a cork borer. The skin was then fully immersed in O.C.T media and frozen in liquid nitrogen. The frozen skin was then sliced horizontally into layers of 200 µm using a cryostatic microtome (Leica CM1900-1-1 cryostatic microtome, Leica Microsystems, Nussloch, Germany). Ciprofloxacin was then extracted from skin specimens with 1 mL acetonitrile via sonication for 4 h and centrifugation for 20 min at 10,000*g* (Eppendorf 5425, UK). All samples were analyzed using the developed reverse-phase HPLC method. The drug distribution resulting from the control was studied in the same manner except that, instead of inserting a ciprofloxacin microneedle array, free gel containing an equivalent amount of ciprofloxacin was placed on top of the skin, followed by the stainless steel weight for consistency.

### Antimicrobial activity: in vitro infection study

**Figure 3:**
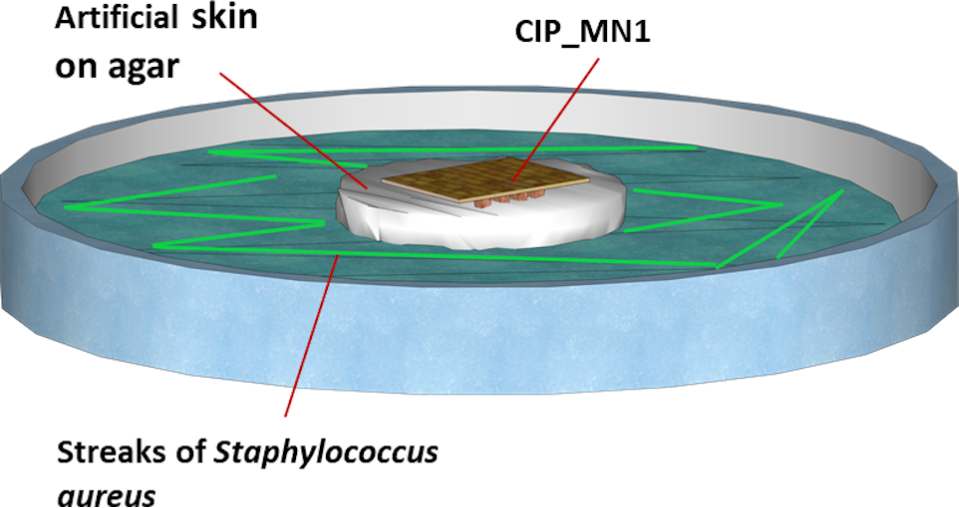
In vitro model for the antimicrobial activity of CIP_MN1. Preincubated inoculum of *S. aureus* is streaked over an agar plate. An artificial agarose-based skin is then put on the top of the agar. A microneedle array of ciprofloxacin, free ciprofloxacin gel, or blank microneedles were put on the surface of the agar. After incubation for 24 h, CIP_MN1, free CIP gel, or blank microneedles were removed, and the zone of inhibition was measured in millimeters using a ruler.

*S. aureus* (ATCC-29213) inoculum equivalent to 0.5 MacFarland (ca. 1 × 10^8^ CFU/mL) turbidity was prepared from an overnight culture. A sterile swab was dipped in the preadjusted inoculum and the excess fluid was expressed. This swab was used to streak the entire surface of nutrient agar plate (Oxoid, UK) (CLSI, 2013). An artificial agarose-based skin (9.5 cm^2^) was put on top of the inoculated agar. Then CIP_MN1, free ciprofloxacin gel, or blank microneedles were put on the surface of the artificial skin. After incubation for 24 h at 37 °C, microneedles loaded with ciprofloxacin (CIP_MN), free gel (with equivalent drug concentration to the microneedles), and blank microneedles (free from drug) were removed, the artificial skin was removed, and the zone of inhibition was measured in millimeters using a ruler (Figure 3).

### Statistical analysis

The results are presented as means ± standard deviation. Statistical comparison between microneedles and free CIP gel, in terms of ciprofloxacin permeation, was made using GraphPad Prism software (ver. 9; GraphPad, Inc. San Diego, CA, USA). A two-tailed Student’s *t*-test was used to compare different pairs of data. One-way and two-way analysis of variance (ANOVA) was used in the skin disposition study to compare the effects of different formulations and/or times of incubation. Rejection of the null hypothesis was considered when *p* < 0.05.

## Results and Discussion

### Microneedles characterization

**Figure 4:**
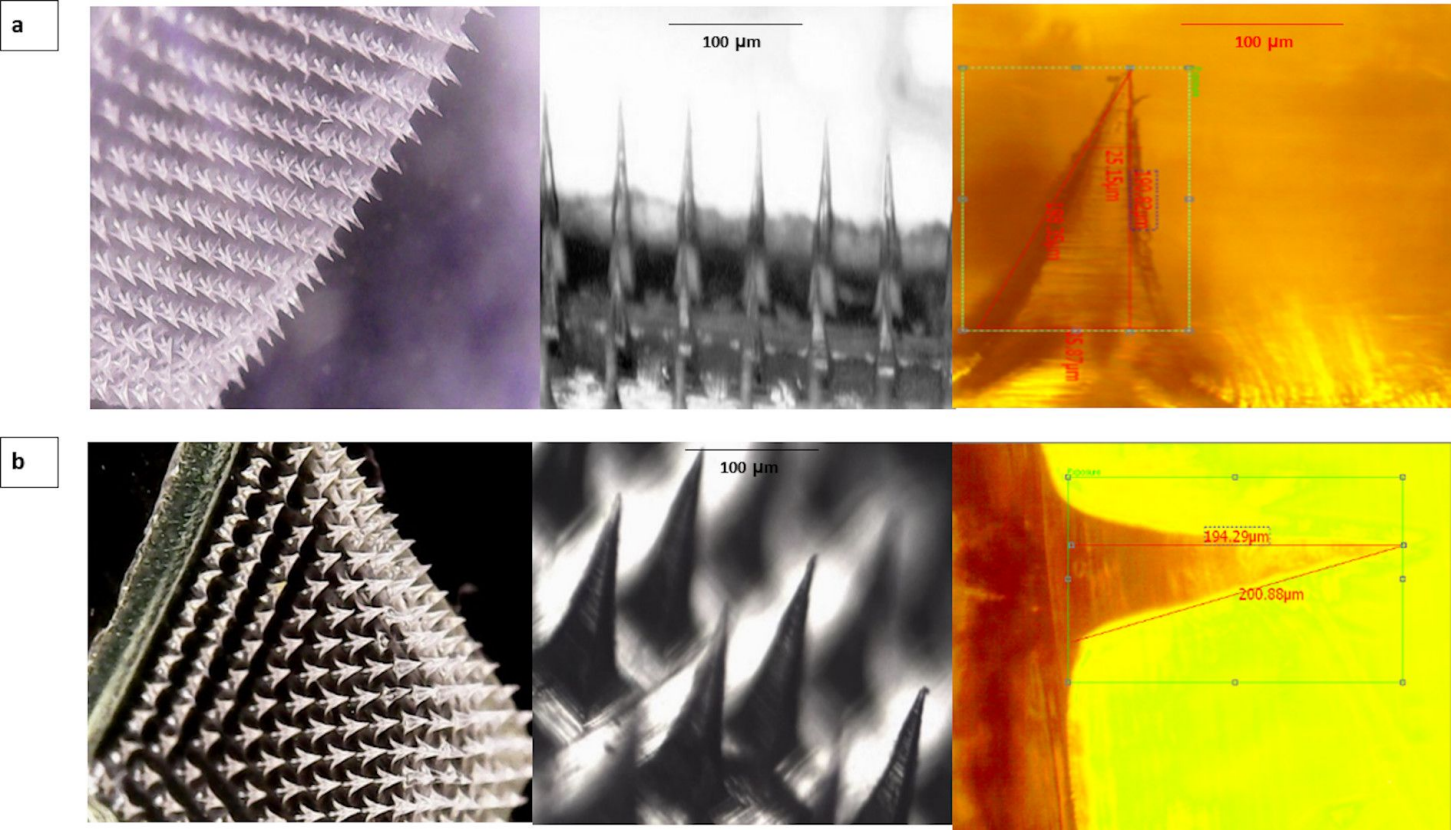
Microneedle visualization for two formulations; (a) CIP_MN1 images under digital light microscope, and (b) CIP_MN2 images under digital light microscope.

The polymeric microneedles were prepared using a mixture of PVA and PVP, or PVA alone. Size and shape were examined using a light microscope. The microneedles showed a pyramidal appearance with an average microneedle height of 188 ± 11 µm and an average square base diameter 49.5 ± 3.4 µm for the formulation of PVA/PVP (9:1) (CIP_MN1). CIP_MN2 showed a microneedle height of 179 ± 16 µm and a square base diameter of 44.2 ± 7.1 µm. As shown in Figure 4, the tips of the microneedles were sharp with less than 5 µm tip diameter. In a previous study, sharp tips with 5 µm tip diameter were required for smooth skin penetration, in contrast to needles with a larger tip diameter (15 µm or more), which showed sudden increase in depth after initial superficial penetration [30]. Moreover, in another study, the square base of the microneedles was shown to cause deeper penetration compared to hexagonal bases [31]. The shape of the microneedles in our study was faceted pyramidal due to the use of the pyramidal PDMS mold in the microneedle preparation. The faceted appearance of the CIP_MN1 and CIP_MN2 microneedles was attributed to the shrinkage of the polymer gel upon drying as suggested by previous studies [32–33]. A similarly faceted shape of microneedles was observed with polymeric microneedles made of chitosan and alginate [34]. Also, a similar microneedle shape was obtained using hydroxypropyl methyl cellulose and PVA as the matrix blend of the microneedles [35]. The CIP content in our study was 170 µg per array for CIP_MN1 and 182 µg per array for CIP_MN2.

### Microneedle penetration capabilities

To assess the skin penetration capabilities of CIP_MN1 and CIP_MN2, we utilized Parafilm as a model to simulate human skin. Parafilm has been proposed as a model that simulates the mechanical properties of porcine skin [36], which is similar in barrier properties to human skin [37]. In a study to assess the insertion depth of microneedles in Parafilm, no significant difference was shown in the insertion depth between porcine skin and Parafilm for insertion forces of 10 and 40 N [36]. In a recent study, Parafilm was also studied as a model that simulates the mechanical and dissolution properties of mammalian skin [38]. Therefore, the ability of the microneedles to penetrate the Parafilm is an indication that microneedles are capable of penetrating human skin. The thickness of the Parafilm is 130 µm. Therefore, the ability of CIP_MN1 to fully puncture Parafilm is an indication that these microneedles can potentially fully penetrate the skin when applied.

**Figure 5:**
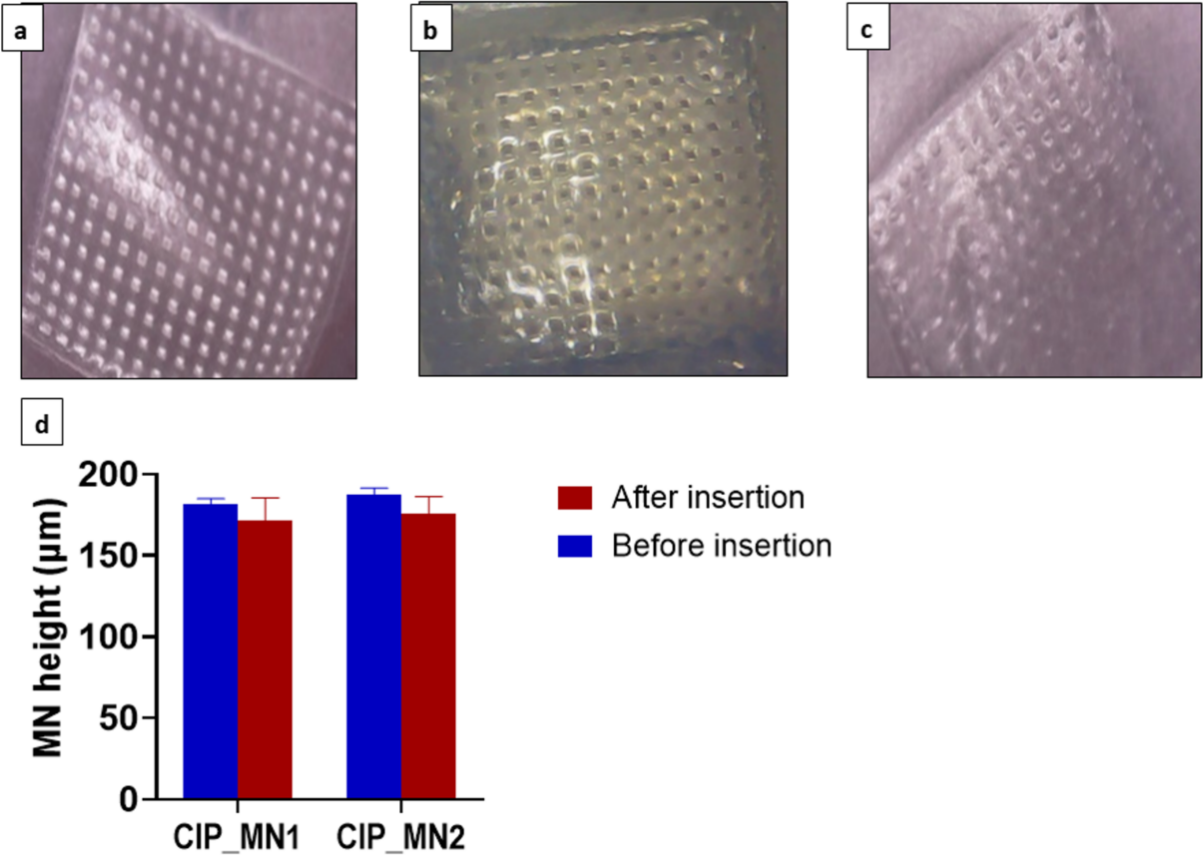
(a) Parafilm penetration with CIP_MN1 microneedles; (b) artificial skin (agarose-based) penetration with CIP_MN1; (c) Parafilm penetration with CIP_MN2 microneedles; (d) heights of CIP_MN1 and CIP_MN2 before and after compression with a thumb press for 30 s, *n* = 3.

CIP_MN1 gave a complete array of perforations for the first layer of Parafilm, which means that the microneedles can reach 130 µm, as shown in Figure 5 and Table 1. However, CIP_MN2 showed significantly fewer perforations compared to the CIP_MN1; the first layer of Parafilm was perforated only by 85 needles. Although PVA hydrogel has shown satisfactory mechanical properties, many studies have shown better mechanical properties of PVA/PVP hydrogels [39–41]. In one study, the tensile strength of PVA hydrogel was increased by 133% after blending with less than 2% w/w PVP [42]. This is due to the formation of relatively strong hydrogen bonds between the hydroxy groups of PVA and the carbonyl groups of PVP in an intertwined network [43]. Therefore, we assumed that CIP_MN1 composed of PVA/PVP hydrogel had greater mechanical strength than CIP_MN2 composed of PVA and penetrated the Parafilm more efficiently. Overall, the results we obtained signify the superiority of the CIP_MN1 formulation, which was chosen to be investigated further.

**Table 1:** Physical characteristics of CIP_MN1 and CIP_MN2, that is, height, diameter, CIP content, and number of pores in Parafilm after insertion, *n* = 3.

	Microneedle height (µm)	Microneedles diameter (µm)	CIP content (µg/array)	Number of pores in Parafilm after insertion

CIP_MN1	188 ± 11	49.5 ± 3.4	170 ± 39	190 ± 3
CIP_MN2	179 ± 16	44.2 ± 7.1	182 ± 45	85 ± 12

Next the penetration capabilities of CIP_MN1 were studied using the in vitro skin model composed of 2.5% agarose gel. Agarose gel is widely used in wound healing for skin regeneration due to its mechanical properties that resemble those of human skin [44–45]. Additionally, using an agarose-based in vitro skin model can help to reduce the skin structure variability to obtain more consistent and reproducible lab results [46]. The 2.5% agarose gel as an artificial skin model revealed penetration of the CIP_MN1 microneedle array, as shown in Figure 5.

The ability of microneedles to withstand the insertion force is crucial in topical and transdermal delivery systems [47]. Compressing the microneedles with a thumb press revealed no significant difference in the height of CIP_MN1 or CIP_MN2 before and after thumb press for 30 s on excised human skin. This indicates that both microneedle formulations withstand the insertion force required for microneedle application on skin. We used thumb press to study the tensile strength of the microneedles since, previously, dissolving polymeric microneedles composed of PVA have been shown to withstand insertion forces equivalent to thumb press [25]. Also, several research studies have used gentle thumb press for in vitro and ex vivo topical/transdermal delivery studies [48–50]. Dissolving microneedles, particularly made of PVP and PVA, have been shown to withstand the required insertion force in skin [51–52].

### Microneedle dissolution in human skin

**Figure 6:**
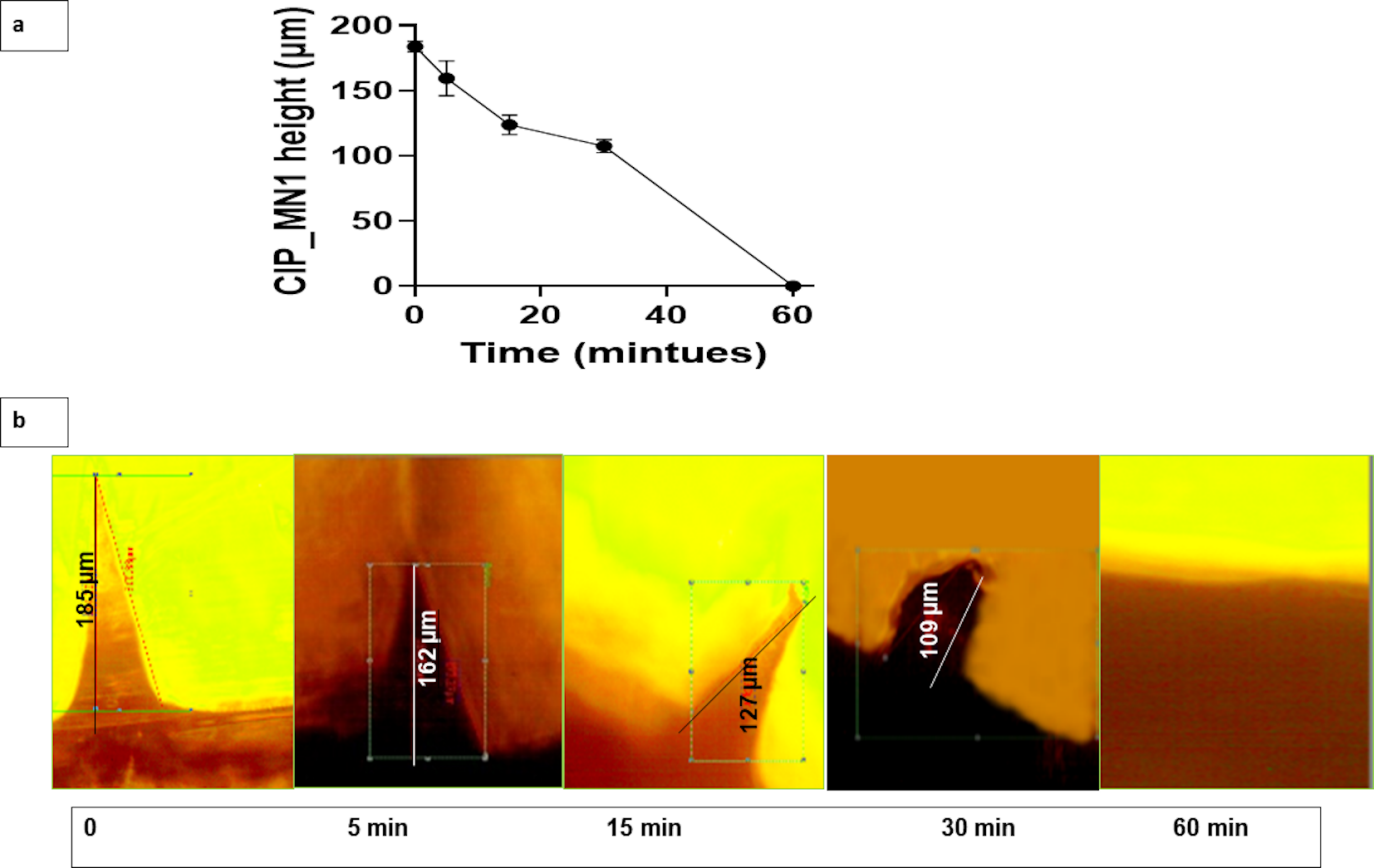
(a) CIP_MN1 dissolution in excised human skin over time (represented by CIP_MN1 height decease with time), *n* = 3; (b) microscopic image of CIP_MN1 at 0, 5, 15, 30, and 60 min after insertion in excised human skin.

The dissolution of CIP_MN1 in human skin showed a gradual decrease in the microneedle length with time. The microneedles required one hour for complete dissolution in the skin as shown in Figure 6. The dissolution of CIP_MN1 was consistent with previous studies. In a recent study on the dissolution of microneedles, PVA-based microneedles have shown to dissolve in porcine skin over a period of 40 min [53]. In another study, PVP/PVA-based microneedles showed to dissolve in the skin of rats within one hour from application [54].

### Skin deposition study

**Figure 7:**
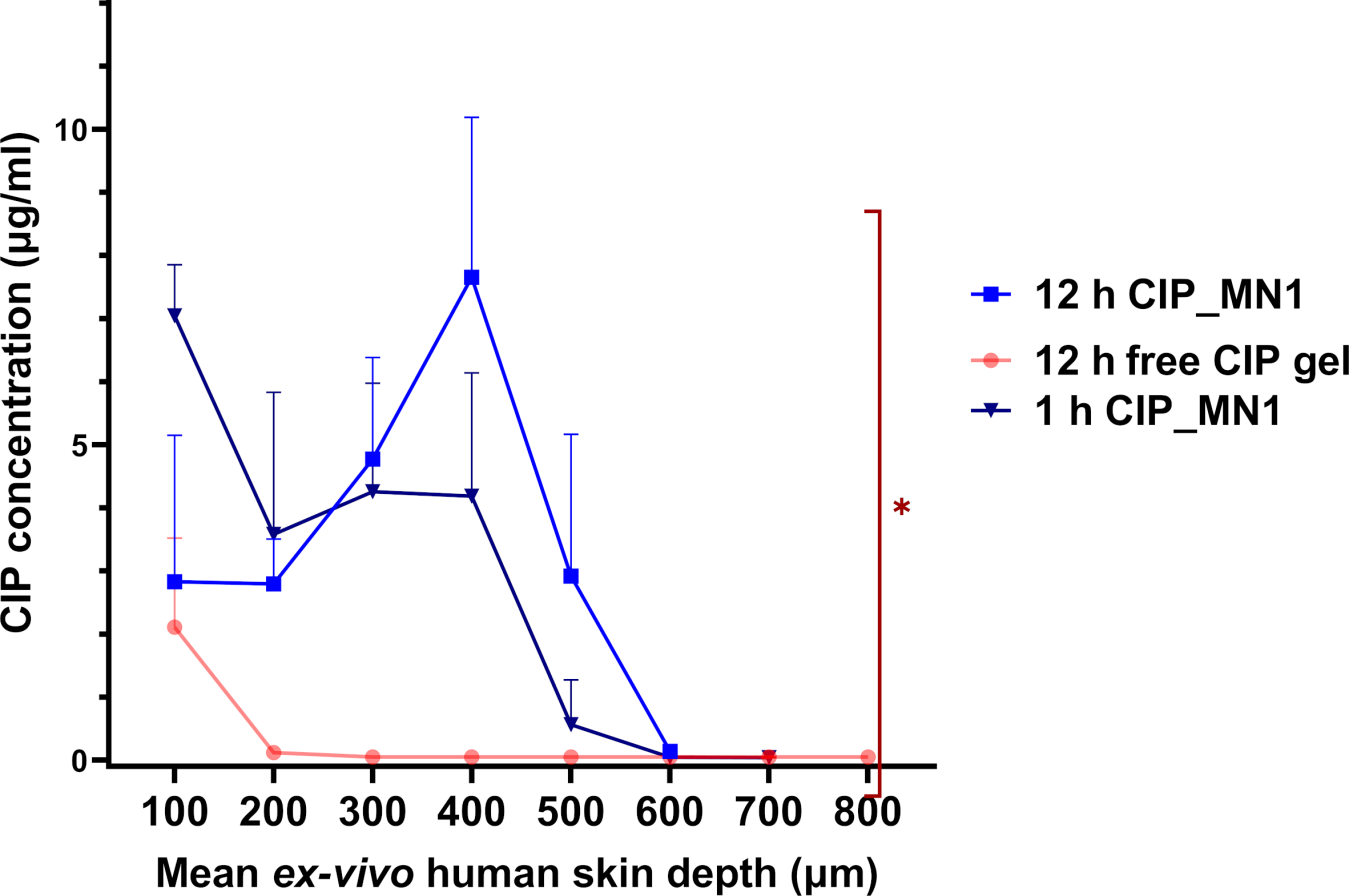
Ciprofloxacin skin deposition from CIP_MN1 after 1 h and 12 h from insertion, and from free gel after 12 h from application, into different depth distances. *P < 0.05 for one-way ANOVA between 12 h CIP_MN1, 1 h CIP_MN1, and free CIP gel; *n* = 4.

One-way ANOVA analysis showed a significant difference in the permeation of ciprofloxacin between the free CIP gel and CIP_MN1, after one hour and after 12 h (*P* value = 0.015). Moreover, the results also showed that the deposition of the incorporated drug into deeper layers could be enhanced by the microneedle delivery system. As shown in Figure 7, ciprofloxacin in CIP_MN1 appeared to migrate to a deeper layer compared to ciprofloxacin in free gel. Ciprofloxacin released from CIP_MN1 permeated to a depth of more than 500 µm compared to less than 200 µm from free gel. The results also showed that ciprofloxacin released from CIP_MN1 appeared to decrease with time in the upper layers and to increase in deeper layers, as shown by comparing the deposition of ciprofloxacin after 1 h and after 12 h. This was evidenced by *p* = 0.044 in a two-way ANOVA analysis for the interaction between skin depth and ciprofloxacin content.

In our skin deposition study, ciprofloxacin was able to migrate deeper than 500 µm after application (CIP_MN1 on excised human skin sample), which means that ciprofloxacin already passed the epidermis and reached the dermis, since the epidermis thickness is generally less than 10–80 µm. Microneedles that can reach deeper than 80 µm can potentially deliver the incorporated drug for the treatment of local and systemic infections [55].

The dermis is a viable layer that is rich in water, blood capillaries, and connective tissues including collagen and elastin [56]. These properties can facilitate the diffusion into the dermis and underlying layers for local and transdermal effects. In a recent study, PVP-based dissolving microneedles were able to deliver sinomenine hydrochloride to a skin depth of 200 µm in rats [57], which is significantly less than the depth that we achieved. In another study showing the efficiency of dissolving microneedles in transdermal delivery, the contraceptive hormone levonorgestrel was released and migrated through the entire skin structure, maintaining a quantifiable plasma concentration for up to 60 days [58].

### Antibacterial activity of CIP_MN1

**Figure 8:**
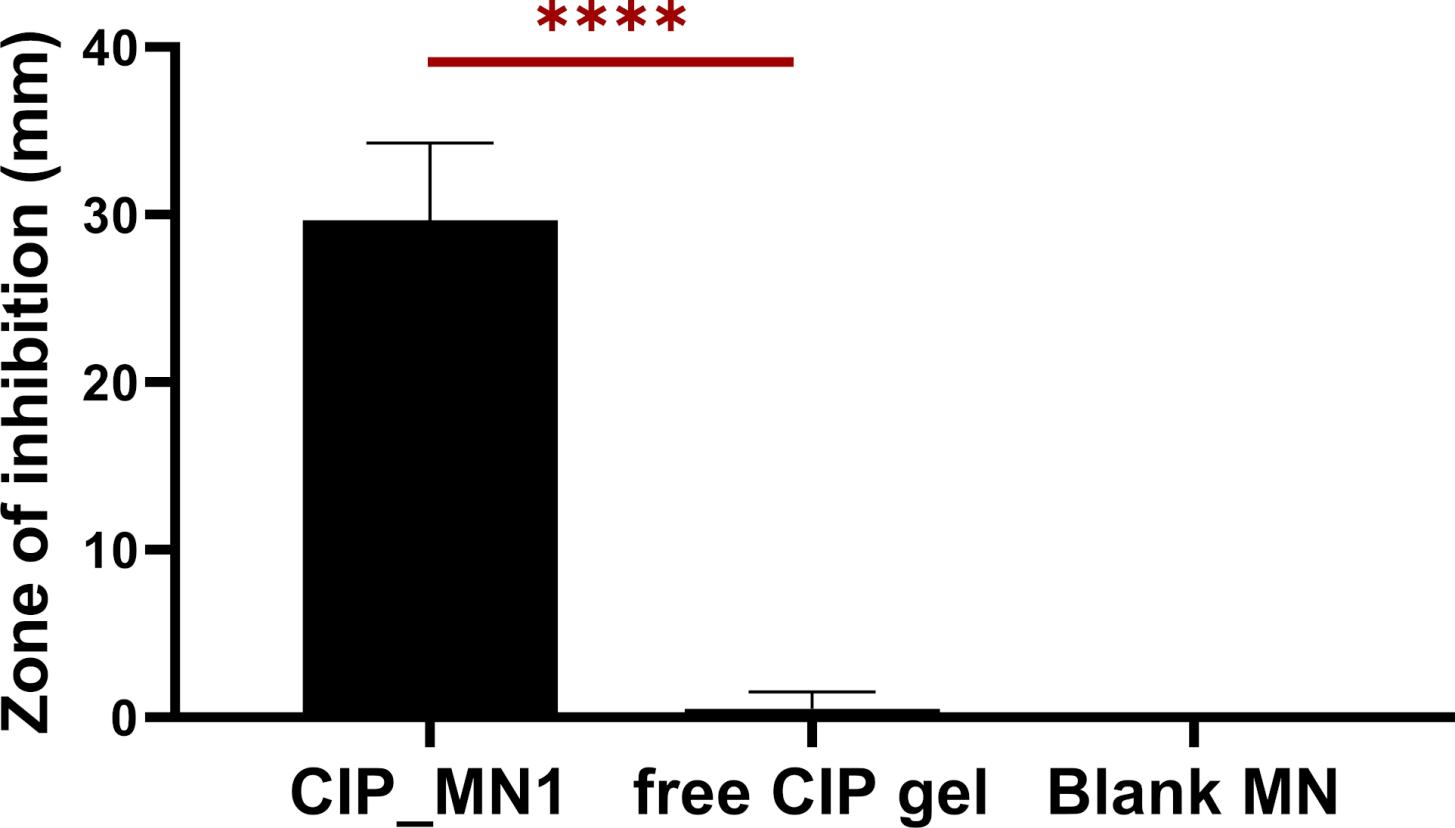
Antibacterial activity of CIP_MN1 applied on an in vitro skin model mounted on *S. aureus*-inoculated agar. ****p < 0.0001; *n* = 3.

Skin models are generally less expensive, provide a solution to the limited availability of fresh human skin samples, and increase safety of handling [59]. Also, utilizing an in vitro skin model with definite bacterial inoculum can reduce the inter- and intra-individual variability in mechanical properties of human skin [60]. After incubation of the artificial skin with the *S. aureus*, a bacterial lawn was obviously formed beneath the untreated skin. As shown in Figure 8, when samples were applied over the artificial skin, a zone of inhibition of 29 mm was observed for CIP_MN1, and one of 2 mm was observed for free ciprofloxacin gel, indicating that ciprofloxacin was able to permeate the artificial skin layer significantly better than the free CIP gel (*p* < 0.0001). No zone of inhibition was observed for the untreated group and the blank microneedles.

CIP-loaded microneedles were studied on three substrates, namely Parafilm, 2.5% agarose, and excised human skin. A summary of the experiments and the principal results is given in Table 2.

**Table 2:** CIP_MN1 and CIP_MN2 penetration capabilities with the rationale of the experiment and the principal outcomes.

Experiment	Substrate	Rationale	Principle results of our study

microneedle penetration capabilities	Parafilm	The penetration depth in Parafilm is similar to the penetration depth in porcine skin and human skin [36–37].	CIP_MN1 showed 190 perforations compared to 85 perforations of CIP_MN2
	2.5% agarose gel in vitro skin model	Agarose gel has shown mechanical properties similar to those of human skin [22–23].	CIP_MN1 (which was used in subsequent skin dissolution and deposition studies) showed complete perforation on 2.5% agarose gel.
antibacterial activity of CIP_MN1 vs free CIP gel	2.5% agarose gel in vitro skin model infected with *Staphylococcus aureus*	Agarose in vitro skin model reduces the inter- and intra-individual variability and is also less expensive and widely available [46].	CIP_MN1 showed a significantly larger zone of inhibition (29 mm) than free gel (2 mm).
dissolution of CIP_MN1 in human skin	excised human skin	Using excised human skin is feasible and requires less strict ethical issue compared to a clinical study. Moreover, excised human skin study provides minimal differences to in vivo conditions [61].	CIP_MN1 showed complete dissolution in the skin one hour after application.
deposition of CIP in the dermal layers			Ciprofloxacin released from CIP_MN1 permeated to a depth of more than 500 µm compared to ciprofloxacin from free gel, which permeated less than 200 µm in depth.

## Conclusion

This study investigated the potential of ciprofloxacin-loaded dissolving microneedles based on PVA and PVP as copolymers for the treatment of *S. aureus* skin infections. The PVA/PVP-based dissolving microneedles were studied regarding drug content, mechanical strength, perforation of Parafilm and an agarose-based skin model, dissolution in excised human skin, and antibacterial activity in the agarose-based skin model infected with *S. aureus*. The results suggest that PVA/PVP microneedles were able to withstand the insertion force and to fully penetrate Parafilm and agarose gel. Moreover, the superiority of this delivery system over conventional administration of free gel in excised human skin and in an in vitro skin infection model was demonstrated. This can be of great benefit for the painless topical/transdermal delivery of antibiotics, overcoming the barrier properties of the skin.
